# Mantra Meditation for Pain Conditions: A Systematic Review and Meta‐Analysis

**DOI:** 10.1155/prm/7796640

**Published:** 2026-10-15

**Authors:** Yao Coitinho Biurra, Colette Naude, Zoe Coitinho Biurra, Anthony Thompson

**Affiliations:** ^1^ Mind-Body Research in Health Laboratory School of Psychology Deakin University Burwood Australia deakin.edu.au; ^2^ School of Psychology Arden University London England UK; ^3^ Universidad de Alicante Alicante Spain ua.es

**Keywords:** chronic pain, mantra, meditation, pain management

## Abstract

**Background:**

Pain is biopsychosocial in nature, and international guidelines now emphasise interdisciplinary treatment. Psychotherapeutic interventions such as mantra meditation (MM) are being increasingly studied for their potential benefits in pain management.

**Objectives:**

To conduct a systematic review and meta‐analysis of the evidence for MM in improving biopsychosocial outcomes in acute and chronic pain conditions.

**Methods:**

A systematic search of MEDLINE, PsycINFO, CINAHL, CENTRAL, EMBASE and Scopus identified controlled clinical trials of MM for acute and chronic pain, with no date limits. Data were synthesised using an inverse‐variance random‐effects meta‐analysis of continuous outcomes with precalculated effect sizes (Hedges’ g).

**Results:**

Seven studies (*n* = 374) met inclusion criteria, with low‐to‐moderate methodological quality. Meta‐analyses showed significant effects of MM versus controls for improving mental quality of life (QoL) and reducing negative mood states. No significant effects were found for pain, physical QoL, depression symptoms, anxiety, stress, psychological well‐being or sleep. However, subgroup analysis indicated significant improvement in depression symptoms for participants with chronic pain compared with acute pain.

**Conclusion:**

Further high‐quality randomised controlled trials are required to determine the efficacy of MM in pain management. Current evidence suggests MM may improve selected psychological outcomes, including mental QoL, negative mood states and depression in chronic pain. These findings indicate that MM has potential as an adjuvant intervention within holistic, interdisciplinary approaches to pain management.

## 1. Introduction

Pain is defined by the International Association for the Study of Pain (IASP) as ‘an unpleasant sensory and emotional experience associated with actual or potential tissue damage, or described in terms of such damage’ [1]. Although criticised for oversimplification [2], pain is usefully categorised into nociceptive, inflammatory and pathological subtypes [3], and as acute or chronic by duration [4]. Chronic pain lasts at least 3 months (e.g., endometriosis, cancer‐related pain) [5], while acute pain involves rapid onset and short duration (e.g., burns, childbirth) [4]. Shared physiological and psychological mechanisms [6] support examining both forms together.

The biopsychosocial model best captures pain’s complexity, integrating biological, psychological and social influences [7, 8]. Individuals with pain frequently experience anxiety, depression, stress, sleep and mood disturbances, impaired function and disrupted relationships [9], driven by inflammation and psychosocial factors such as poor mental health or limited support [10]. These erode quality of life (QoL). Global prevalence is 28% across 52 countries (*n* = 199,862) [11], generating an annual economic burden of $560–635 billion in medical costs, lost productivity and disability support [12]. Such personal, societal and financial costs demand effective interventions.

Pharmacological treatment predominates, beginning with over‐the‐counter analgesics before diagnosis [13, 14] and progressing to tailored regimens of opioids [13, 15] and NSAIDs [15, 16]. Long‐term NSAID use carries cardiovascular and gastrointestinal risks [17]. Opioids exacerbate dependence, fuel the current crisis [18] and nearly double all‐cause mortality in older adults compared with NSAIDs [19–21].

Recognising these limitations and pain’s psychosocial burden, the UK National Institute for Health and Care Excellence (NICE) advocates multidisciplinary management [22]. This emphasises psychotherapeutic interventions delivered by mental health professionals [23–25], sometimes prioritised over pharmacotherapy or surgery [26, 27].

Established psychological therapies include cognitive behavioural therapy (CBT) [28], acceptance and commitment therapy (ACT) [28], hypnotherapy [29] and mindfulness meditation [30]. CBT has the strongest evidence, significantly reducing pain and disability [31–34], as shown in a review of 75 trials (*N* = 9401) [34]. ACT improves distress and function, particularly in chronic pain [35, 36]. Hypnotherapy benefits irritable bowel syndrome and emotional well‐being [37–40]. Mindfulness meditation stands out for its accessibility—no therapist required, fully self‐administered [41]—and consistent reductions in pain intensity and distress across populations [42–48], aligning with demand for nonpharmacological self‐management.

Mantra meditation (MM), a concentrative practice centred on repeated mantras [49, 50], is emerging as a promising alternative. While MM improves mental health [50–52] and spiritual well‐being [53], its role in pain management is still nascent.

Rooted in Hinduism, Buddhism [54] and Zoroastrianism [55], ‘mantra’ derives from Sanskrit roots denoting mental repetition that ‘saves’ or protects from suffering [56]. Mantras—sounds, words or phrases—may be breath‐synchronised [57], chanted aloud or silently [58] or simply listened to [57]. Examples include Transcendental Meditation, Kirtan Kriya and Sahaj Samadhi [59–62]. MM uniquely engages linguistic pathways rather than somatic focus [63], a mechanism paralleled in speech‐language pathology by melodic intonation therapy, where repetitive, rhythmic vocal production recruits dedicated linguistic‐motor networks in speech rehabilitation [64, 65], and by neuroscientific evidence that sustained verbal recitation is associated with structural changes in memory‐ and language‐related brain regions [66]. Traditional science holds that efficacy peaks when mantras are ‘chetan’—transmitted by a perfected teacher [57, 67–69].

Neuroimaging reveals MM’s mechanisms. One study showed 40%–50% reductions in thalamic, prefrontal and global brain responses to pain, with modest anterior cingulate attenuation, dampening emotional and motivational processing [70]. Another study (*n* = 16) identified a signature of heightened attention (elevated anterior cingulate and dorsolateral prefrontal flow) paired with reduced arousal (decreased pons and cerebellar activity), confirming low effort and high feasibility [71]. Global interest is rising [51]; 1.6% of US adults (3.6 million) practised MM over 12 months, only 0.3% below mindfulness [72].

Despite this, no systematic review has synthesised MM’s effects on pain. Given the field’s early stage, broad inclusion criteria are recommended to capture available data comprehensively [50, 73, 74]. This review therefore evaluates MM interventions for acute and chronic pain conditions, across adult and paediatric populations, and all controlled study designs. It addresses four research questions:

Can MM improve self‐reported pain intensity in individuals with pain conditions? Can MM improve QoL in people with pain conditions? Can MM reduce symptoms of depression, anxiety and stress in people with pain conditions? Can MM enhance mood states, psychological well‐being and sleep quality in people with pain conditions?

## 2. Method

The present review followed the Preferred Reporting Items for Systematic Reviews and Meta‐Analyses (PRISMA) guidelines [75]. The review protocol was registered prospectively on the International Prospective Register of Systematic Reviews (PROSPERO) on the 10th of October 2024, with reference number CRD42024598688 [76]. This project, due to being a review, was exempted from ethics approval.

### 2.1. Study Selection

#### 2.1.1. Eligibility Criteria

Studies were eligible if they were English‐language, peer‐reviewed, controlled trials (randomised or non‐randomised) involving human participants of any age or sex experiencing acute [4] or chronic [5] pain. Interventions had to consist of MM as the primary component, delivered in any format or duration (face‐to‐face or online); adjunctive breathing exercises or brief visualisations were permitted, but MM could not be combined with any other active intervention. Comparators could be any non‐MM control (wait‐list, treatment‐as‐usual or active). Outcomes were self‐reported using validated instruments and included pain intensity (primary), QoL, depression, anxiety, stress, negative mood, psychological well‐being and sleep quality.

Studies were excluded if they were grey literature, conference abstracts, uncontrolled or did not evaluate an MM intervention.

### 2.2. Search Strategy and Study Selection

A comprehensive search was conducted across Medline Complete, APA PsycINFO, CINAHL, Cochrane CENTRAL, EMBASE and Scopus (see Appendix A for sample strategy (see Table A1)). Reference lists of included studies and relevant reviews were hand‐searched. No date limits were applied; searches were restricted to English‐language publications and completed on 15 October 2024, and updated on 10^th^ October 2025.

Records were imported into Covidence [77]. Titles and abstracts were screened independently by two reviewers (Yao Coitinho Biurra, Zoe Coitinho Biurra), with conflicts resolved by a third (Anthony Thompson). Full‐text articles were assessed independently by the same two reviewers, with discrepancies resolved by discussion.

### 2.3. Data Extraction

Data were extracted independently by two reviewers ((Yao Coitinho Biurra, Zoe Coitinho Biurra) into a pre‐piloted Excel spreadsheet (Appendix B), with conflicts resolved by AT. Extracted items included study characteristics (authors, year, country, design, setting), participant details (sample size, age, pain condition), intervention features (MM type, duration, delivery mode, comparator), outcome measures and all means and standard deviations (or standard errors) at every time point. A data extraction checklist is provided in Appendix C (see Table A2).

### 2.4. Data Synthesis and Statistical Analysis

Comparable outcomes were synthesised using inverse‐variance random‐effects meta‐analyses in IBM SPSS [78], following the intention‐to‐treat principle. Postintervention data (first follow‐up after intervention end) were converted to Hedges’ g to accommodate small samples and varying reporting formats, using the George Mason University online effect‐size calculator [79]. Effect sizes were interpreted as small (g = 0.15), medium (0.40) or large (0.75) [80]. Heterogeneity was assessed with I^2^ (< 25% low, 50% moderate, 75% high) [81].

Acute and chronic pain studies were pooled because of the limited evidence base [50, 73, 74]. Subgroup analyses by pain type (acute vs chronic) were performed only when ≥ 2 studies were available per category. When multiple measures existed for one outcome, the most appropriate was selected. A narrative synthesis complemented the quantitative results.

### 2.5. Quality Assessment

Risk of bias was assessed independently by two reviewers (Yao Coitinho Biurra, Zoe Coitinho Biurra) using the Cochrane Risk of Bias Tool Version 1 [82], with disagreements resolved by discussion. The tool evaluates seven domains (random sequence generation, allocation concealment, blinding of participants and personnel, blinding of outcome assessment, incomplete outcome data, selective reporting and other bias), each rated as low, high or unclear risk.

## 3. Results

### 3.1. Study Selection From the Literature Search

All stages of screening are presented in the PRISMA flow diagram below [75] (see Figure 1). A total of 2588 studies were selected for potential inclusion, and 369 duplicates were removed. A further 2194 records were excluded after screening titles and abstracts against the selection criteria. The remaining 25 full‐text records were further assessed for eligibility, and another 18 were excluded, mostly due to the wrong study design or population. Discrepancies between reviewers were resolved through discussion. The remaining seven studies met the selection criteria and were included in the final meta‐analyses.

**FIGURE 1:**
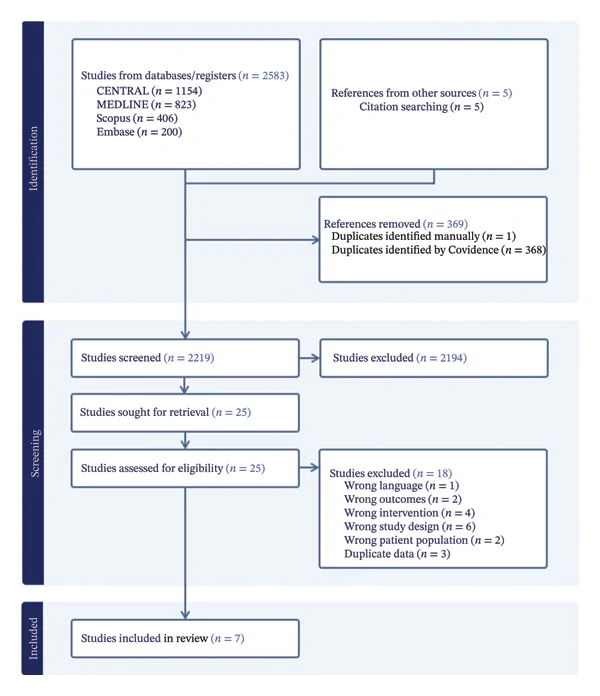
PRISMA diagram of study selection [75].

#### 3.1.1. Study Characteristics

Table 1 summarises the key characteristics of the studies included in this review. Seven RCTs were included, with sample sizes ranging from 22 [86] to 99 [85]. Four studies were conducted in the United States [60, 83, 84, 86], one in Germany [85], one in India [59] and one in the Netherlands [87].

**TABLE 1:** Summary characteristics of included randomised controlled trials.

First author (year) (country)	Final sample size (intervention: control)	Sex % (male: Female)	Type of pain	Condition	Mean age (STDV) (years)	Intervention	Delivery	Control intervention	Duration (weeks)	Follow‐up (months after treatment)	Outcome variables
Jayadevappa, Johnson [83] (USA)	13:10	39:61	Chronic	Congestive heart failure	64.14 (9.13)	Transcendental Meditation	In‐person training. At‐home practice.	Health education	12	3, 6	QWB, PSS, LHFQ, 6‐MWT, CES‐D, BNP, Cortisol
Wachholtz and Pargament [84] (USA)	22:20	12:88	Acute	Migraines	19.21 (7.09)	Spiritual (mantra) meditation	In‐person training. At‐home practice.	Progressive muscle relaxation	4	1	Headache severity/frequency, CES‐D, TAI, headache self‐efficacy, various spiritual outcomes
Yogitha, Nagarathna [59] (India)	30:30	53:47	Acute	Common neck pain	41.63 (14.82)	Yogic Sound Resonance	Pre‐recorded audio	Supine rest	1.5	0.33	PAS, Tenderness, NDS, Flexion, Extension, RLF, LLF, RLR, LLR, STAI, BPS, BPD, Pulse
Jeitler, Brunnhuber [85] (Germany)	45:44	18:22	Chronic	Chronic neck pain	49.64 (10.22)	Jyoti Meditation	Meditation group, or at‐home practice after in‐person training.	Exercise	4	1, 2	Pain VAS, bothersomeness VAS, NPAD, CPS, SF‐36, CES‐D, STAI,
Innes, Selfe [60] (USA)	30:30	15:85	Acute	Alzheimer’s disease	60.58 (1.01)	Kirtan Kriya	In‐person training, at‐home practice with pre‐recorded audio.	Music listening	12	3, 6	PSS, PMS, PWBS, PSQI, SF‐36
Innes, Selfe [86] (USA)	11:11	32:68	Chronic	Knee osteoarthritis	58.46 (1.37)	Mantra Meditation	In‐person training, at‐home practice with pre‐recorded audio.	Music listening	8	2	KOOS, Pain NRS, PSS, PSQI, PMS, PWBS, SF‐36
Jong, Boers [87] (The Netherlands)	37:41	23:77	Acute	Primary headaches	13.39 (2.61)	Transcendental Meditation	In‐person training. At‐home practice.	Progressive muscle relaxation	12	3, 9	Headache frequency, adequate relief, RCADS, PCQ, CSI

*Note*: STDV, standard deviation; QWB, Quality of Well‐Being survey; LHFQ: Minnesota Living with Heart Failure Questionnaire, 6‐MWT: six‐minute walk test, SF‐36: 36‐Item Short Form Health Survey, CES‐D: Center for Epidemiologic Studies Depression Scale, NPAD: Neck Pain and Disability Index, CPS: Cohen Perceived Stress Scale, KOOS: Knee Injury and Osteoarthritis Outcome Score. Acute/chronic classification follows ICHD‐3 convention for migraine (episodic attacks vs. ≥ 15 headache days/month for > 3 months) [5], and Bone and Joint Decade Task Force criteria for neck pain (< 3 months vs. > 3 months) [88].

Abbreviations: BNP, brain natriuretic peptide; CSI, Children Somatisation Inventory; PMS, Profile of Mood States; PCQ, Pain Coping Questionnaire; PSQI, Pittsburgh Sleep Quality Index; PSS, Perceived Stress Scale; PWBS, Psychological Well‐Being Scale; RCADS, Revised Children’s Anxiety and Depression Scale; STAI, State‐Trait Anxiety Inventory; TAI, Trait Anxiety Inventory; USA, United States of America.

#### 3.1.2. Participant Characteristics

There were a total of 374 participants across 7 studies, with 188 participants in the intervention conditions and 186 in the control conditions. Mean ages ranged from 13.39 (STDV = 2.61) [87] to 64.14 (STDV = 9.13) [83] years. Six studies included adult populations, while only one [87] included a paediatric population (*n* = 78). While six studies included female‐dominant samples (74.38% females vs 25.62% males), only one was male‐dominant (53%) [59]. Further participant characteristics are presented in Table 1.

#### 3.1.3. Intervention Characteristics

Two studies [83, 87] implemented Transcendental Meditation, two [84, 86] applied a general MM, one [84] used ‘spiritual’ mantras, one [60] employed Kirtan Kriya, one [59] utilised Yogic Sound Resonance, and one [85] investigated Jyoti Meditation. The intervention delivery methods varied: three studies [83, 84, 87] provided group‐based in‐person training, where the intervention itself was later self‐directed by participants at home, based on what they learned during training. Three studies [59, 60, 86] followed a similar delivery format, but included additional pre‐recorded audios for guided at‐home sessions. Lastly, one study [85] provided the intervention via in‐person group sessions, but also allowed for at‐home practice. Intervention durations ranged from 1.5 weeks [59] to 12 weeks [60, 83, 87], while follow‐up timeframes ranged from 3 weeks [59] to 9 months [87] (see Table 1 for further details).

#### 3.1.4. Control Characteristics

As presented in Table 1, all the studies employed parallel controlled group designs, with all but one study [59] using an active control. Active control interventions differed across studies: Three studies [84, 85, 87] compared the intervention to progressive muscle relaxation (PMR), two [60, 86] used music listening, and one [83] used health education, while the nonactive control study [59] employed nonguided supine rest.

#### 3.1.5. Outcomes and Measures

All included studies measured both physiological and psychological outcomes (see Table 1 for further details). The measures used for the meta‐analyses were as follows: The PSS was used for stress, the PMS for mood, PWBS for psychological well‐being, CES‐D and RCADS for symptoms of depression, STAI and RCADS for symptoms of anxiety, SF‐36 for physical and mental QoL and the PSQI for sleep quality; meanwhile, NRS, VAS and SF‐36 (bodily pain subscale) were used for pain intensity.

### 3.2. Risk of Bias

The results of the risk‐of‐bias appraisals for the included studies are presented in Table 2. Across the studies, the risk‐of‐bias domains that were appraised as having a high or unclear risk of bias were the blinding of participants (performance bias) and blinding of outcome assessment (detection bias), as well as in the ‘other sources of bias’ category, mostly due to small sample sizes [83, 86] or lack of symmetry in the control treatment [85]. All other domains were appraised as having a low or mixed (low, high, or unclear across different domains) risk of bias.

**TABLE 2:** Risk‐of‐bias appraisal for included studies.

	Jayadevappa, Johnson [83]	Wachholtz and Pargament [84]	Yogitha, Nagarathna [59]	Jeitler, Brunnhuber [85]	Innes, Selfe [60]	Innes, Selfe [86]	Jong, Boers [87]
Random sequence generation (selection bias)	Low	Low	Low	Low	Low	Low	Low
Allocation concealment (selection bias)	Low	Low	Low	Low	Low	Low	Low
Blinding of participants (performance bias)	Low	High	High	Low	Low	Unclear	Unclear
Blinding of outcome assessment (detection bias)	Low	Unclear	High	Low	Low	Low	Unclear
Incomplete outcome data (attrition bias)	Low	Low	Low	High	Low	Low	Low
Selective reporting (reporting bias)	Low	Low	Low	Low	Low	Low	Low
Other sources of bias	High	Low	Low	High	Low	High	Low

### 3.3. Pain Intensity

Six out of seven studies (*N* = 296) [59, 60, 83–86] provided a measure of pain intensity. To ensure consistency in interpreting the results across the variety of measures used (which interpreted results in opposing directions), effect sizes underwent a directional adjustment by multiplying the NRS and VAS results by −1. This adjustment was necessary because the SF‐36 pain subscale scores increased with reducing pain intensity, while VAS and NRS scores decreased with reducing pain intensity. This modification allowed for a uniform directionality of effect sizes, facilitating the accurate interpretation of the meta‐analysis.

Overall, no significant difference in pain intensity was observed between the MM and control groups posttreatment, g = 0.07 [‐0.18,0.33], *Z* = 0.57, *p* = 0.57 (Figure 2). Heterogeneity was low, *I* ^2^ = 18%.

**FIGURE 2:**
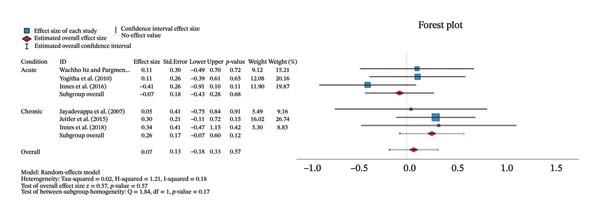
Forest plot of MM versus control groups for the outcome of pain intensity postintervention. Positive values favour MM.

### 3.4. Health‐Related QoL

#### 3.4.1. Physical Component

Three out of seven studies (*N* = 171) [60, 85, 86] measured physical QoL using the SF‐36 physical component. Overall, no significant difference in physical QoL was observed between the MM and control groups posttreatment, g = 0.10 [−0.2, 0.39], *Z* = 0.63, *p* = 0.53 (Figure 3). Heterogeneity was extremely low, *I* ^2^ = 0%.

**FIGURE 3:**
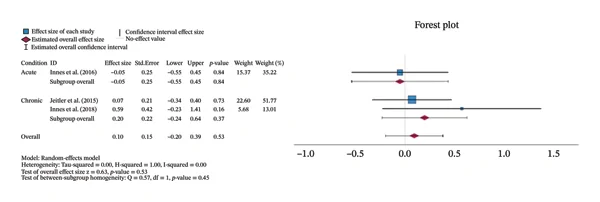
Forest plot of MM versus control groups for the outcome of physical QoL postintervention.

#### 3.4.2. Mental Component

Three out of seven studies (*N* = 171) [60, 85, 86] measured mental QoL using the SF‐36 mental component. A significant increase in mental QoL postintervention was found for participants in the MM versus control groups, indicative of a moderate effect [80], g = 0.38 [0.08, 0.68], *Z* = 2.48, *p* = 0.01 (Figure 4). Heterogeneity was extremely low, *I* ^2^ = 0%.

**FIGURE 4:**
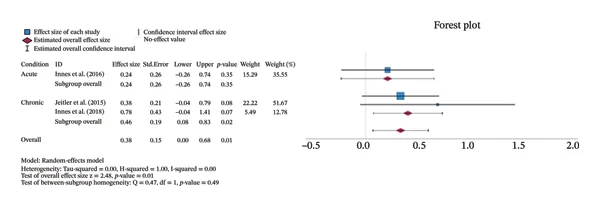
Forest plot of MM versus control groups for the outcome of mental QoL postintervention.

### 3.5. Depression

Four out of seven studies (*N* = 232) [83–85] measured symptoms of depression using the RCADS [87] and the CES‐D. Overall, no significant difference in depression was observed between the MM and control groups posttreatment, g = −0.11 (−0.57, 0.35), *Z* = −0.46 and *p* = 0.64 (Figure 5). Heterogeneity was high, *I* ^2^ = 65%.

**FIGURE 5:**
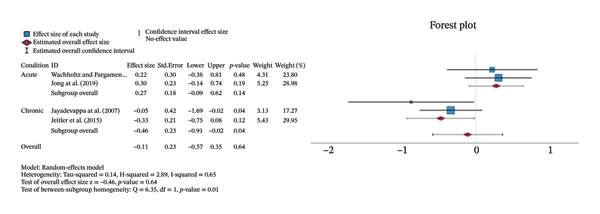
Forest plot of MM versus control groups for the outcome of depression postintervention.

Further subgroup analysis was performed, where only studies utilising participants with chronic conditions were included (*N* = 112) [83, 85]. A significant reduction in depression was found for participants in the MM versus control groups posttreatment, indicative of a moderate effect [80], g = −0.46 [−0.91, −0.02], *Z* = −2.04 and *p* = 0.04 (Figure 5). Heterogeneity was low, *I* ^2^ = 18%.

### 3.6. State Anxiety

Two out of seven studies (*N* = 149) [59, 85] measured state anxiety using the STAI. No significant difference in state anxiety was observed between the MM and control groups posttreatment, g = −0.56 [−1.124, 0.001], *Z* = −1.96 and *p* = 0.05 (Figure 6). Heterogeneity was high, *I* ^2^ = 65%. Given the small number of studies (2), these results should be read with caution.

**FIGURE 6:**
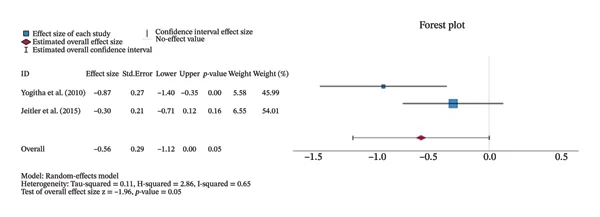
Forest plot of MM versus control groups for the outcome of state anxiety postintervention.

### 3.7. Trait Anxiety

Two out of seven studies (*N* = 131) [84, 85] measured trait anxiety using the STAI. No significant difference in trait anxiety was observed between the MM and control groups posttreatment, g = −0.13 [−0.47, 0.21], *Z* = −0.74 and *p* = 0.46 (Figure 7). Heterogeneity was low, − = 0%. Given the small number of studies (2), these results should be read with caution.

**FIGURE 7:**
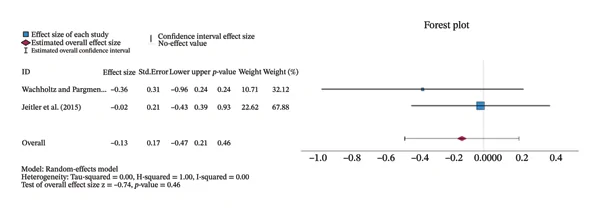
Forest plot of MM versus control groups for the outcome of trait anxiety postintervention.

### 3.8. Mood States

Two out of seven studies (*N* = 82) [60, 86] measured results for (negative) mood states using the PMS. A significant reduction in mood states was found for participants in the MM versus control groups posttreatment, indicative of a moderate effect [80], g = −0.55 [−0.98, −0.11], *Z* = −2.48 and *p* = 0.01 (Figure 8). Heterogeneity was low, *I* ^2^ = 0%. Given the small number of studies (2), these results should be read with caution.

**FIGURE 8:**
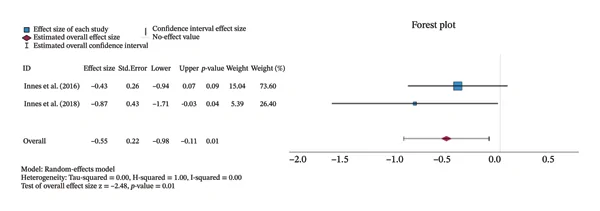
Forest plot of MM versus control groups for the outcome of mood states postintervention.

### 3.9. Psychological Well‐Being

Two out of seven studies (*N* = 82) [60, 86] reported results for psychological well‐being using the PWBS. No significant difference in psychological well‐being was observed between the MM and control groups posttreatment, g = 0.43 [−0.004, 0.856], *Z* = 1.94, *p* = 0.05 (Figure 9). Heterogeneity was low, *I* ^2^ = 0%. Given the small number of studies (2), these results should be read with caution.

**FIGURE 9:**
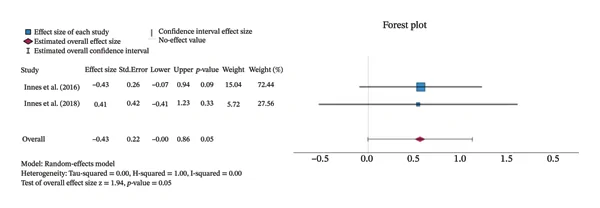
Forest plot of MM versus control groups for the outcome of psychological well‐being postintervention.

### 3.10. Stress

Four out of seven studies (*N* = 194) [60, 83, 85, 86] measured stress, using the PSS. No significant difference in stress was observed between the MM and control groups posttreatment, g = −0.19 [−0.47, 0.09], *Z* = −1.34 and *p* = 0.18 (Figure 10). Heterogeneity was low, *I* ^2^ = 0%.

**FIGURE 10:**
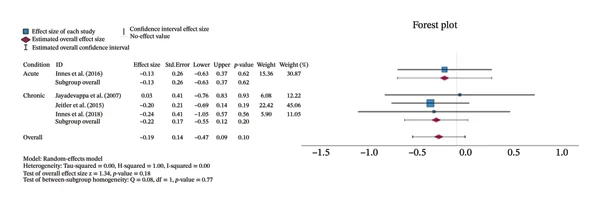
Forest plot of MM versus control groups for the outcome of stress postintervention.

### 3.11. Sleep Quality

Two out of seven studies (*N* = 82) [60, 86] reported results for sleep quality, using the SQI. No significant difference in sleep quality was observed between the MM and control groups posttreatment, g = −0.38 [−1.21, 0.45], *Z* = −0.91, *p* = 0.36 (Figure 11). Heterogeneity was substantial, *I* ^2^ = 66%. Given the small number of studies (2), these results should be read with caution.

**FIGURE 11:**
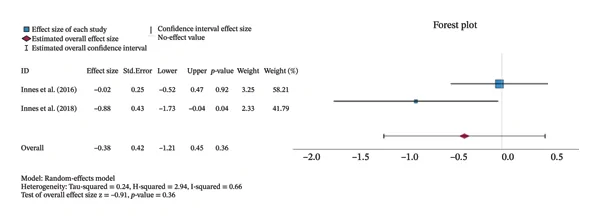
Forest plot of MM versus control groups for the outcome of sleep quality.

## 4. Discussion

This meta‐analysis is the first to synthesise the evidence for MM in improving biopsychosocial outcomes among individuals with acute and chronic pain. MM is defined as the focused repetition of a specific word, sound, or phrase—silently or aloud—engaging a distinct linguistic pathway rather than bodily sensations or external objects [63]. Neuroimaging studies have demonstrated that MM produces unique patterns of brain activity, significantly attenuates emotional and motivational responses to pain (40%–50% reduction in thalamic, prefrontal and global responses) and operates with minimal cognitive effort, supporting its feasibility as a scalable intervention [70, 71].

Given the embryonic stage of MM research, broad inclusion criteria were deliberately adopted [50, 73, 74]. The review therefore encompassed both acute and chronic pain conditions, adult and paediatric populations and a wide range of self‐reported biopsychosocial outcomes: pain intensity (primary), mental and physical QoL, psychological well‐being, negative mood states, state and trait anxiety, depression and sleep quality.

Overall, the findings provide preliminary but encouraging evidence that MM can improve selected biopsychosocial domains in pain populations. A statistically significant improvement emerged for mental QoL, consistent with a large systematic review (*k* = 51) demonstrating small‐to‐moderate gains in general‐population mental health [51]. Negative mood states were also significantly reduced postintervention, extending the limited narrative evidence from an earlier review (*k* = 37) [50].

For depression, the overall pooled effect was nonsignificant; however, subgroup analysis restricted to chronic pain revealed a significant reduction. This pattern is plausible given the markedly higher prevalence and severity of depressive symptoms in chronic versus acute pain [89–91], affording greater scope for therapeutic change [46, 92]. The result aligns with two prior meta‐analyses confirming MM’s benefits for depression in nonclinical samples [50, 51].

In contrast, MM produced no significant effect on pain intensity—the first meta‐analytic evaluation of this outcome. Heterogeneity in pain measures and diagnostic categories (e.g., headache versus osteoarthritis) likely contributed to an underpowered analysis. Pain’s inherently biopsychosocial nature further complicates detection of specific intervention effects. Similarly, no significant improvement occurred in physical QoL, an unsurprising finding given its strong correlation with pain intensity [93] and the absence of pain reduction.

Previous reviews reported benefits of MM for sleep quality [51], psychological well‐being [94, 95] and both state and trait anxiety [50]. The present analyses, however, yielded null results for stress, anxiety, psychological well‐being and sleep. Each of these outcomes rested on only two small studies, several with substantial heterogeneity, rendering the meta‐analyses underpowered. Likewise, although earlier general‐population reviews (*k* = 51 and *k* = 37) found stress reduction [50, 51], the current null finding may reflect the review’s focus on individuals with clinical pain rather than healthy adults; stress in pain populations is inextricably linked to physiological burden, potentially requiring more targeted or intensive intervention [46, 92].

Considerable heterogeneity existed across MM protocols. Most employed Sanskrit mantras, but several used English [84–86]; session durations ranged from 1.5 to 12 weeks; and one protocol involved multiple sequential mantras [59]. Delivery modes were largely consistent (in‐person or home‐based), yet few studies explicitly followed traditional ‘chetan’ transmission—impartation by a perfected teacher—which mantra theory posits is essential for maximal efficacy [57, 67–69]. These variations preclude firm conclusions about optimal parameters and highlight the urgent need for standardised, evidence‐based protocols.

### 4.1. Strengths and Limitations

Strengths include prospective registration on PROSPERO [76], strict adherence to PRISMA guidelines [75] and dual independent review at every stage of screening, data extraction and quality appraisal, with third‐reviewer resolution of discrepancies. The broad yet clearly justified scope enabled the first biopsychosocial evaluation of MM applied to a physical health condition, distinguishing this work from prior mental‐health‐only reviews.

Limitations are nevertheless important. Only seven studies met inclusion criteria, each with modest sample sizes; not all outcomes were reported in every trial, further reducing statistical power. Pooling paediatric and adult samples alongside acute and chronic pain conditions introduced clinical heterogeneity. Intervention variability (type, duration, language, number of mantras) and the absence of traditional mantra impartation likely attenuated observable effects. Adherence to home practice and adverse events were rarely reported, limiting safety and feasibility conclusions. Most studies used active but nonspecific controls (e.g., PMR, rest, education) that failed to match MM’s core components or benchmark against gold‐standard psychotherapies such as CBT or ACT. Risk‐of‐bias ratings were generally moderate, driven primarily by inadequate blinding and small samples. Collectively, these factors necessitate cautious interpretation.

### 4.2. Future Research

Future trials should employ active controls that closely replicate MM’s attentional and repetitive elements (e.g., mindfulness meditation or music‐assisted relaxation [60, 86]) to maintain blinding and isolate specific effects. Inclusion of gold‐standard comparators (CBT, ACT) is also recommended. Traditional mantra transmission should be incorporated, readily achievable through pre‐recorded sessions delivered by expert practitioners. Studies must be adequately powered, use homogeneous protocols and outcome measures, monitor adherence rigorously, report safety data and include extended follow‐up.

Once the evidence base expands, subsequent systematic reviews should conduct subgroup analyses by pain type (nociceptive, neuropathic, inflammatory), age group and MM characteristics (session length, delivery mode, mantra language, syllable count) to identify dose–response relationships and optimal protocols [53]. Separate meta‐analyses for acute versus chronic pain and paediatric versus adult populations will further refine clinical recommendations.

A related but mechanistically distinct paradigm—group or synchronous chanting, which engages social entrainment through joint vocalisation rather than the solitary, linguistic–attentional focus characteristic of individual MM practice—represents a promising avenue for future pain research [96, 97].

### 4.3. Clinical Implications

Current NICE guidelines advocate multidisciplinary pain management that prioritises psychotherapeutic interventions, sometimes over pharmacotherapy or surgery [22–27]. Although MM did not reduce pain intensity, it produced meaningful gains in mental QoL and negative mood states—outcomes that are highly valued by patients and strongly associated with improved functioning and treatment adherence.

MM is brief to learn, low‐burden, portable, equipment‐free and adaptable to any cultural or religious context. It can be delivered in‐person or via scalable pre‐recorded formats, offering cost‐effective access. These attributes position MM as a practical adjunct within existing pain services, either as a standalone self‐management tool or integrated with conventional care, thereby advancing truly holistic, patient‐centred pain management.

## 5. Conclusion

This first meta‐analysis of MM for pain indicates preliminary benefits for mental QoL, negative mood and (in chronic pain) depression, despite no effect on pain intensity itself. The small number of modest‐quality studies precludes definitive conclusions. Nevertheless, MM’s accessibility and feasibility suggest it may serve as a valuable low‐intensity adjunct in multidisciplinary pain programmes. High‐quality, adequately powered randomised trials using standardised protocols, appropriate controls and rigorous reporting are now urgently required to clarify its role and optimise its application.

## Funding

This study did not receive any specific funding from public, commercial or not‐for‐profit agencies. Open access publishing facilitated by Deakin University, as part of the Wiley‐Deakin University agreement via the Council of Australasian University Librarians.

## Disclosure

The present manuscript was made available as a preprint at [98] https://www.researchsquare.com/article/rs-9536601/v1.

## Conflicts of Interest

The authors declare no conflicts of interest.

## Data Availability

Data sharing is not applicable to this article as no datasets were generated or analysed during the current study.

## Appendix A Search Strategy

**TABLE A1:** Search strategy used across Medline Complete, APA PsycINFO, CINAHL, Cochrane CENTRAL, EMBASE, and Scopus.

Mantra Meditation	S1	TI mantra^∗^ OR AB mantra^∗^
	S2	TI mantra meditation OR AB mantra meditation
	S3	TI kriya OR AB kriya
	S4	TI kirtan kriya OR AB kirtan kriya
	S5	TI transcendental meditation OR AB transcendental meditation
	S6	TI vedic meditation OR AB vedic meditation
	S7	TI sahaj samadhi OR AB sahaj samadhi
	S8	TI meditative phrases OR AB meditative phrases
	S9	TI phrase repetition OR AB phrase repetition
	S10	TI spiritual meditation OR AB spiritual meditation
	S11	TI mantra repetition OR AB mantra repetition
	S12	TI japa OR AB japa
	S13	TI bija mantra OR AB bija mantra
	S14	TI aum OR AB aum
	S15	TI om OR AB om
	S16	TI yogic sound resonance OR AB yogic sound resonance
	S17	TI mind sound resonance OR AB mind sound resonance
	S18	TI mantra^∗^ yoga OR AB mantra^∗^ yoga
	S19	TI brahmanada OR AB brahmanada
	S20	TI divine sound meditation OR AB divine sound meditation
	S21	TI divine brahmnaad OR AB divine brahmnaad
	S22	TI nada yoga OR AB nada yoga
	S23	TI mantrochar OR AB mantrochar
	S24	TI healing mantra OR AB healing mantra
	S25	TI TM OR AB TM
	S26	TI affirmation^∗^ OR AB affirmation^∗^
	S27	TI Word^∗^ repetition OR AB Word^∗^ repetition
	S28	TI affirmation repetition OR AB affirmation repetition
	S29	TI affirmation meditation OR AB affirmation meditation
	S30	S1 OR S2 OR S3 OR S4 OR S5 OR S6 OR S7 OR S8 OR S9 OR S10 OR S11 OR S12 OR S13 OR S14 OR S15 OR S16 OR S17 OR S18 OR S19 OR S20 OR S21 OR S22 OR S23 OR S24 OR S25 OR S26 OR S27 OR S28 OR S29

Pain Conditions	S31	TI osteoarthritis OR AB osteoarthritis
	S32	TI rheumatoid arthritis OR AB rheumatoid arthritis
	S33	TI fibromyalgia OR AB fibromyalgia
	S34	TI chronic lower back pain OR AB chronic lower back pain
	S35	TI chronic neck pain OR AB chronic neck pain
	S36	TI chronic joint pain OR AB chronic joint pain
	S37	TI myofascial pain syndrome OR AB myofascial pain syndrome
	S38	TI tendinitis OR AB tendinitis
	S39	TI bursitis OR AB bursitis
	S40	TI frozen shoulder OR AB frozen shoulder
	S41	TI adhesive capsulitis OR AB adhesive capsulitis
	S42	TI chronic pelvic girdle pain OR AB chronic pelvic girdle pain
	S43	TI scoliosis OR AB scoliosis
	S44	TI ankylosing spondylitis OR AB ankylosing spondylitis
	S45	TI temporomandibular joint disorders OR AB temporomandibular joint disorders
	S46	TI TMJ disorders OR AB TMJ disorders
	S47	TI acute pain OR AB acute pain
	S48	TI diabetic neuropathy OR AB diabetic neuropathy
	S49	TI postherpetic neuralgia OR AB postherpetic neuralgia
	S50	TI trigeminal neuralgia OR AB trigeminal neuralgia
	S51	TI phantom limb pain OR AB phantom limb pain
	S52	TI peripheral neuropathy OR AB peripheral neuropathy
	S53	TI sciatica OR AB sciatica
	S54	TI complex regional pain syndrome OR AB complex regional pain syndrome
	S55	TI CRPS OR AB CRPS
	S56	TI carpal tunnel syndrome OR AB carpal tunnel syndrome
	S57	TI radiculopathy OR AB radiculopathy
	S58	TI central post‐stroke pain OR AB central post‐stroke pain
	S59	TI multiple sclerosis OR AB multiple sclerosis
	S60	TI spinal cord injury OR AB spinal cord injury
	S61	TI chronic migraine OR AB chronic migraine
	S62	TI tension headaches OR AB tension headaches
	S63	TI cluster headaches OR AB cluster headaches
	S64	TI cervicogenic headaches OR AB cervicogenic headaches
	S65	TI sinus headaches OR AB sinus headaches
	S66	TI occipital neuralgia OR AB occipital neuralgia
	S67	TI chronic daily headaches OR AB chronic daily headaches
	S68	TI facial pain syndromes OR AB facial pain syndromes
	S69	TI chronic pelvic pain OR AB chronic pelvic pain
	S70	TI endometriosis OR AB endometriosis
	S71	TI interstitial cystitis OR AB interstitial cystitis
	S72	TI painful bladder syndrome OR AB painful bladder syndrome
	S73	TI irritable bowel syndrome OR AB irritable bowel syndrome
	S74	TI IBS OR AB IBS
	S75	TI chronic prostatitis OR AB chronic prostatitis
	S76	TI chronic pelvic pain syndrome OR AB chronic pelvic pain syndrome
	S77	TI CP/CPPS OR AB CP/CPPS
	S78	TI pelvic floor dysfunction OR AB pelvic floor dysfunction
	S79	TI vulvodynia OR AB vulvodynia
	S80	TI dysmenorrhea OR AB dysmenorrhea
	S81	TI menstrual pain OR AB menstrual pain
	S82	TI cancer‐related pain OR AB cancer‐related pain
	S83	TI chemotherapy‐induced peripheral neuropathy OR AB chemotherapy‐induced peripheral neuropathy
	S84	TI radiation‐induced pain OR AB radiation‐induced pain
	S85	TI post‐surgical cancer pain OR AB post‐surgical cancer pain
	S86	TI bone cancer pain OR AB bone cancer pain
	S87	TI tumor‐related pain OR AB tumor‐related pain
	S88	TI post‐surgical chronic pain OR AB post‐surgical chronic pain
	S89	TI post‐mastectomy pain syndrome OR AB post‐mastectomy pain syndrome
	S90	TI post‐amputation pain OR AB post‐amputation pain
	S91	TI post‐thoracotomy pain syndrome OR AB post‐thoracotomy pain syndrome
	S92	TI post‐traumatic pain OR AB post‐traumatic pain
	S93	TI chronic pain after spinal surgery OR AB chronic pain after spinal surgery
	S94	TI chronic pancreatitis OR AB chronic pancreatitis
	S95	TI chronic appendicitis OR AB chronic appendicitis
	S96	TI gallbladder disease OR AB gallbladder disease
	S97	TI chronic kidney pain OR AB chronic kidney pain
	S98	TI kidney stones OR AB kidney stones
	S99	TI chronic liver pain OR AB chronic liver pain
	S100	TI chronic abdominal pain OR AB chronic abdominal pain
	S101	TI lupus‐related pain OR AB lupus‐related pain
	S102	TI psoriatic arthritis OR AB psoriatic arthritis
	S103	TI inflammatory bowel disease OR AB inflammatory bowel disease
	S104	TI IBD OR AB IBD
	S105	TI Crohn’s disease‐related pain OR AB Crohn’s disease‐related pain
	S106	TI ulcerative colitis‐related pain OR AB ulcerative colitis‐related pain
	S107	TI chronic fatigue syndrome OR AB chronic fatigue syndrome
	S108	TI myalgic encephalomyelitis OR AB myalgic encephalomyelitis
	S109	TI Ehlers‐Danlos syndrome‐related pain OR AB Ehlers‐Danlos syndrome‐related pain
	S110	TI sickle cell disease‐related pain OR AB sickle cell disease‐related pain
	S111	TI polymyalgia rheumatica OR AB polymyalgia rheumatica
	S112	TI hereditary sensory neuropathy OR AB hereditary sensory neuropathy
	S113	TI chronic Lyme disease pain OR AB chronic Lyme disease pain
	S114	TI hypersensitivity disorders OR AB hypersensitivity disorders
	S115	TI chronic venous insufficiency pain OR AB chronic venous insufficiency pain
	S116	TI thoracic outlet syndrome OR AB thoracic outlet syndrome
	S117	TI non‐specific chronic pain OR AB non‐specific chronic pain
	S118	S31 OR S32 OR S33 OR S34 OR S35 OR S36 OR S37 OR S38 OR S39 OR S40 OR S41 OR S42 OR S43 OR S44 OR S45 OR S46 OR S47 OR S48 OR S49 OR S50 OR S51 OR S52 OR S53 OR S54 OR S55 OR S56 OR S57 OR S58 OR S59 OR S60 OR S61 OR S62 OR S63 OR S64 OR S65 OR S66 OR S67 OR S68 OR S69 OR S70 OR S71 OR S72 OR S73 OR S74 OR S75 OR S76 OR S77 OR S78 OR S79 OR S80 OR S81 OR S82 OR S83 OR S84 OR S85 OR S86 OR S87 OR S88 OR S89 OR S90 OR S91 OR S92 OR S93 OR S94 OR S95 OR S96 OR S97 OR S98 OR S99 OR S100 OR S101 OR S102 OR S103 OR S104 OR S105 OR S106 OR S107 OR S108 OR S109 OR S110 OR S111 OR S112 OR S113 OR S114 OR S115 OR S116 OR S117

Final search	S119	S30 AND S118

## Appendix C PRISMA Search and Data Extraction Checklist [71].

**TABLE A2:** PRISMA 2020 search and data extraction checklist.

Methods		
Eligibility criteria	5	Specify the inclusion and exclusion criteria for the review and how studies were grouped for the syntheses.
Information sources	6	Specify all databases, registers, websites, organisations, reference lists and other sources searched or consulted to identify studies. Specify the date when each source was last searched or consulted.
Search strategy	7	Present the full search strategies for all databases, registers and websites, including any filters and limits used.
Selection process	8	Specify the methods used to decide whether a study met the inclusion criteria of the review, including how many reviewers screened each record and each report retrieved, whether they worked independently, and, if applicable, details of automation tools used in the process.
Data collection process	9	Specify the methods used to collect data from reports, including how many reviewers collected data from each report, whether they worked independently, any processes for obtaining or confirming data from study investigators, and, if applicable, details of automation tools used in the process.
Data items	10a	List and define all outcomes for which data were sought. Specify whether all results that were compatible with each outcome domain in each study were sought (e.g., for all measures, time points, analyses), and, if not, the methods used to decide which results to collect.
	10b	List and define all other variables for which data were sought (e.g., participant and intervention characteristics, funding sources). Describe any assumptions made about any missing or unclear information.

Study risk‐of‐bias assessment	11	Specify the methods used to assess risk of bias in the included studies, including details of the tool(s) used, how many reviewers assessed each study and whether they worked independently, and, if applicable, details of automation tools used in the process.
Effect measures	12	Specify for each outcome the effect measure(s) (e.g., risk ratio, mean difference) used in the synthesis or presentation of results.
Synthesis methods	13a	Describe the processes used to decide which studies were eligible for each synthesis (e.g., tabulating the study intervention characteristics and comparing against the planned groups for each synthesis (Item #5)).
	13b	Describe any methods required to prepare the data for presentation or synthesis, such as handling of missing summary statistics, or data conversions.
	13c	Describe any methods used to tabulate or visually display results of individual studies and syntheses.
	13d	Describe any methods used to synthesise results and provide a rationale for the choice(s). If meta‐analysis was performed, describe the model(s), method(s) to identify the presence and extent of statistical heterogeneity, and software package(s) used.
	13e	Describe any methods used to explore possible causes of heterogeneity among study results (e.g., subgroup analysis, metaregression).
	13f	Describe any sensitivity analyses conducted to assess robustness of the synthesised results.

Reporting bias assessment	14	Describe any methods used to assess risk of bias due to missing results in a synthesis (arising from reporting biases).
Certainty assessment	15	Describe any methods used to assess certainty (or confidence) in the body of evidence for an outcome.
